# Electroacupuncture for the treatment of frozen shoulder: A protocol for systematic review and meta-analysis

**DOI:** 10.1097/MD.0000000000028179

**Published:** 2021-12-23

**Authors:** Jeong-Weon Heo, Jeong-Hun Jo, Jung-Ju Lee, Hee Kang, Tae-Young Choi, Myeong-Soo Lee, Jong-In Kim

**Affiliations:** a Department of Clinical Korean Medicine, Graduate School, Kyung Hee University, Seoul, Republic of Korea; b Humanitas College, Kyung Hee University, Yongin, Republic of Korea; c Clinical Research Division, Korea Institute of Oriental Medicine, Daejeon, Republic of Korea.

**Keywords:** electroacupuncture, frozen shoulder, protocol, systematic review

## Abstract

**Background:**

Electroacupuncture has been reported to successfully control pain. Currently, no systematic reviews of the effects of electroacupuncture on frozen shoulder patients have been performed. This review aims to provide current evidence on the efficacy of electroacupuncture for the management of pain in frozen shoulder.

**Methods and analyses:**

Eleven databases will be searched from their inception: PubMed, AMED, EMBASE, the Cochrane Library, 6 Korean medical databases, and 1 Chinese medical database. Study selection, data extraction, and assessment will be performed independently by 2 researchers. Risk of bias will be assessed using the Cochrane risk of bias assessment tool.

**Ethics and dissemination:**

Ethical approvals and patient consent are not required because the meta-analysis will be based on published research. This systematic review will be published in a peer-reviewed journal and disseminated both electronically and in print. The review will be updated to inform and guide health care practice and policy.

**Trial registration number:**

PROSPERO 2021 CRD42021247090.

## 1 Introduction

### 1.1 Description of the condition

Frozen shoulder (FS) is a multifactorial disease in which patients present with limited active and passive shoulder movement.^[1]^ FS is characterized by 3 phases: the freezing phase, frozen phase, and thawing phase. The time duration for FS to completely heal varies from 1 to 4 years.^[2]^

FS can be classified as a primary (or idiopathic) condition but is often associated with other diseases and conditions. Patients with diabetes mellitus are at greater risk of developing FS with a prevalence of 10% to 20%.^[3]^

### 1.2 Description of the intervention

Acupuncture has been reported to be effective for the treatment of FS. Many studies have been performed to evaluate the effectiveness of acupuncture for FS, including its ability to relieve pain.^[4,5]^

Electroacupuncture is based on acupuncture. Specifically, a small current passes between pairs of acupuncture needles. The same acupoints are inserted with needles, and several pairs of needles can be simultaneously stimulated during the treatment. Based on standard operation procedures, electroacupuncture is safe, and it is easy to keep the current under control and not exceed the endurance of patients.

### 1.3 How the intervention might work

The mechanism of action underlying the effects of electroacupuncture on controlling pain, so-called “electroacupuncture analgesia”, involves nervous system activation and the induction of bioactive chemicals. Electroacupuncture activates the nervous system and alleviates both sensory and affective inflammatory pain. Electroacupuncture blocks pain by activating a variety of bioactive chemicals through peripheral, spinal, and supraspinal mechanisms. These bioactive chemicals include opioids, which desensitize peripheral nociceptors and reduce proinflammatory cytokines peripherally and in the spinal cord as well as serotonin and norepinephrine, which decrease spinal *N*-methyl-D-aspartate receptor subunit GluN1 phosphorylation.^[6]^

### 1.4 Why a review of the intervention is important

The use of an intraarticular corticosteroid for patients with FS is associated with greater benefits compared with other interventions, leading to improved range of motion and pain reduction.^[7]^ However, the effect is of limited duration.^[8]^ Acupuncture can have a lasting effect on FS with minimal effects to the human body. A meta-analysis on acupuncture for FS has already been performed,^[9]^ but we think that electroacupuncture and manual acupuncture treatments are not interchangeable and thus must be separately identified for accurate study. In systematic review studies, the blending of manual acupuncture and electroacupuncture is detrimental to the homogeneity of studies on acupuncture effects.^[10]^ To our knowledge, no systematic reviews on the effects of electroacupuncture treatments on FS have been reported.

### 1.5 Objective

This review aims to systematically evaluate the evidence on the safety and effectiveness of electroacupuncture for controlling pain in patients with FS from randomized controlled trials (RCTs).

## 2 Methods

### 2.1 Study registration

This protocol has been registered on PROSPERO 2021 CRD (http://www.crd.york.ac.uk/PROSPERO/display_record.php?ID=CRD42021247090).

### 2.2 Criteria for considering studies for this review

#### 2.2.1 Types of studies

Prospective RCTs will be included. We will exclude observational, cohort, case-control, case series, qualitative studies, uncontrolled trials, and laboratory studies. No language restriction will be imposed.

#### 2.2.2 Types of participants

We will include patients with FS of any age, sex, and race. We will only include studies in which an external set of criteria was used to screen participants for the condition.

#### 2.2.3 Types of interventions and controls

Studies that have evaluated any type of invasive acupuncture with electrical stimulation will be included. Control interventions may include treatments, such as general conventional care (drugs, exercise, etc), sham treatment (interventions mimicking “true” electroacupuncture/true treatment but deviating in at least one aspect considered important by electroacupuncture theory, such as skin penetration or correct point location), or waiting list care. We will also include trials that have compared electroacupuncture plus another active treatment with the same other active treatment alone. We will exclude RCTs in which one type of electroacupuncture is compared with a different type of electroacupuncture.

#### 2.2.4 Type of outcome measures

##### 2.2.4.1 Primary outcomes

1) Pain intensity: visual analog scale, numerical rating scale.

##### 2.2.4.2 Secondary outcomes

1) Functional status/disability: Constant–Murley Score, range of motion,
2) Total effective rate,
3) Adverse events.

### 2.3 Search method to identify studies

#### 2.3.1 Electronic searches

Electronic databases searched from their inception will include MEDLINE, EMBASE, the Cochrane Central Register of Controlled Trials (CENTRAL), CINII, 6 Korean databases (KoreaMed, the Korean Traditional Knowledge Portal, Oriental Medicine Advanced Searching Integrated System, DBpia, the Research Information Service System, and the Korean Studies Information Service System), and 1 Chinese database (China National Knowledge Infrastructure). Articles identified through reference lists of the included studies and relevant systematic reviews will be considered for inclusion based on study title. Study selection will be documented and summarized in a preferred reporting items for systematic reviews and meta-analysis-compliant flow chart (http://www.prisma-statement.org).

#### 2.3.2 Search for other resources

The authors will scan the reference lists and retrieve additional studies. In addition, the authors will search the WHO International Clinical Trials Registry Platform (http://apps.who.int/trialsearch/) and Google Scholar (http://scholar.google.co.kr/). The ClinicalTrials.gov registry (http://clinicaltrials.gov/) will be searched for any unpublished trials.

#### 2.3.3 Search strategy

Our search strategy will include main keywords, such as “electroacupuncture”, “frozen shoulder”, “adhesive capsulitis”, and “periarthritis of shoulder”, in English, Chinese, Japanese, and Korean.

### 2.4 Data collection, extraction, and assessment

#### 2.4.1 Study selection

Two reviewers (JWH and JHJ) will independently screen the titles and abstracts for searched studies, assess the criteria for study selection and record their decisions according to predefined criteria. Another reviewer (JIK) will resolve disagreements in study selection. The study selection process will be documented and summarized in a preferred reporting items for systematic reviews and meta-analysis flow diagram.

#### 2.4.2 Data extraction

All articles will be read by 2 independent reviewers (JWH and JHJ) who will extract data from the articles according to predefined criteria. The extracted data will include the author name(s), year of publication, country, sample size, age and sex of the participants, electroacupuncture intervention, control intervention, main outcomes, and adverse effects. The extracted data will be tabulated for future analysis. We will use the grading of recommendations assessment, development and evaluation (GRADE) software to determine the quality of evidence based on the Cochrane Handbook for Systematic Reviews of Interventions to create a summary of findings table.^[11]^ When reported data are insufficient or unclear, an author will contact the first author or corresponding authors by e-mail or telephone to request missing data or clarify data.

#### 2.4.3 Assessment of risk of bias

Quality assessment will be performed using the tool for “risk of bias” assessment from the Cochrane Handbook for Systematic Reviews of Interventions.^[12]^ The following characteristics will be assessed: random sequence generation, allocation concealment, blinding of participants and personnel, blinding of outcome assessment, incomplete outcome data, selective outcome reporting, and other sources of bias (we will evaluate baseline imbalance). This review will use “L, U, and H” as keys for these evaluations, where “Low” (L) indicates a low risk of bias, “Unclear” (U) indicates that the risk of bias is uncertain, and “High” (H) indicates a high risk of bias. Disagreements will be resolved by discussion among all authors. Information regarding the risk of bias assessment for the included studies will be summarized in a table, and the results and implications will be critically discussed.

### 2.5 Data analysis

All statistical analyses will be conducted using the Cochrane Collaboration software Review Manager (RevMan), v.5.4.1 for Windows (The Nordic Cochrane Center, Copenhagen, Denmark). Differences between the intervention and control groups will be assessed. In the analysis of clinical efficacy, categorical data will be assessed in terms of risk ratios, and continuous data will be assessed in terms of mean difference (MD). Categorical and continuous variables will be expressed as efficacy values with 95% confidence intervals. In cases of outcome variables with different scales, standardized MD will be used instead of weighted MD. If we detected heterogeneity (defined by the results of tests of heterogeneity with *P* < .1 by the chi-square test and Higgins I^2^ ≥ 50%), subgroup analyses were performed to determine the cause of clinical heterogeneity. A random effects model will be used to assess combined effect sizes from efficacy variables because substantial clinical heterogeneity is expected across the included studies based on the diversity of interventions, study design, and other conditions. Publication bias will be assessed using funnel plots and Egger regression method.^[13]^ If missing data are detected, we will request any missing or incomplete information from the investigators of the original study.

Subgroup analysis will be conducted according to different types of electroacupuncture frequencies, treatment sessions (<10 times vs >10 times), and the design of the trial (electroacupuncture vs conventional treatment; electroacupuncture vs manual acupuncture; electroacupuncture vs sham electroacupuncture; electroacupuncture combined with other active treatments vs other active treatments alone). Where appropriate, sensitivity analysis will be performed to evaluate the robustness of the meta-analysis results. To verify consistency with other meta-analyses and meta-regression, the primary quality assessment will be a binary measure of allocation concealment.^[14]^

## 3 Ethics and dissemination

Ethical approvals and patient consent are not required because the meta-analysis will be based on published research. This systematic review will be published in a peer-reviewed journal and disseminated both electronically and in print. The review will be updated to inform and guide health care practice and policy.

## 4 Discussion

To date, there has been no systematic review on the use of electroacupuncture for controlling pain in FS. The results of this systematic review evaluating the evidence on the safety and effectiveness of electroacupuncture for treating pain in FS from RCTs may be utilized by clinicians to manage pain associated with FS.

## Author contributions

Contribution of authors: The protocol of a review was drafted by all authors. The search strategy was established by JWH, and JHJ. Copies of studies will be obtained by JWH, TYC, and HK. Selection of the studies for inclusion will be performed by JWH, MSL, and JIK. JIK will act as an arbiter at the study selection stage. Extraction of data from studies will be conducted by JWH and JHJ. JWH and JIK will enter data into RevMan 5.4.1. All authors will interpret the results. The final review will be drafted and revised by all authors. The review will be updated by all authors.

**Conceptualization:** Jong-In Kim.

**Data curation:** Jeong-Weon Heo, Jeong-Hun Jo.

**Formal analysis:** Jeong-Weon Heo, Jeong-Hun Jo, Jung-Ju Lee, Jong-In Kim.

**Investigation:** Jeong-Weon Heo, Jeong-Hun Jo, Hee Kang, Tae-Young Choi.

**Methodology:** Jeong-Weon Heo, Jeong-Hun Jo.

**Project administration:** Myeong-Soo Lee, Jong-In Kim.

**Resources:** Jung-Ju Lee, Hee Kang.

**Software:** Jeong-Weon Heo, Jong-In Kim.

**Supervision:** Hee Kang, Tae-Young Choi, Myeong-Soo Lee, Jong-In Kim.

**Writing – original draft:** Jeong-Weon Heo.

**Writing – review & editing:** Jeong-Weon Heo, Jeong-Hun Jo, Jung-Ju Lee, Hee Kang, Tae-Young Choi, Myeong-Soo Lee, Jong-In Kim.
